# Supplementary material for: Severe COVID-19 patients have impaired plasmacytoid dendritic cell-mediated control of SARS-CoV-2
Source: Nat Commun. 2023 Feb 8;14:694. doi: 10.1038/s41467-023-36140-9 (PMC9907212; doi:10.1038/s41467-023-36140-9)
Supplement: Supplementary file 4 — Source Data [file 41467_2023_36140_MOESM4_ESM.zip › Source data/Venet Fig 2b_pDCs_def/2022-04-28_Thu_16-52-11_GradientBoostClassifier_3/Confusion_matrix_Downsampling 3.pdf]

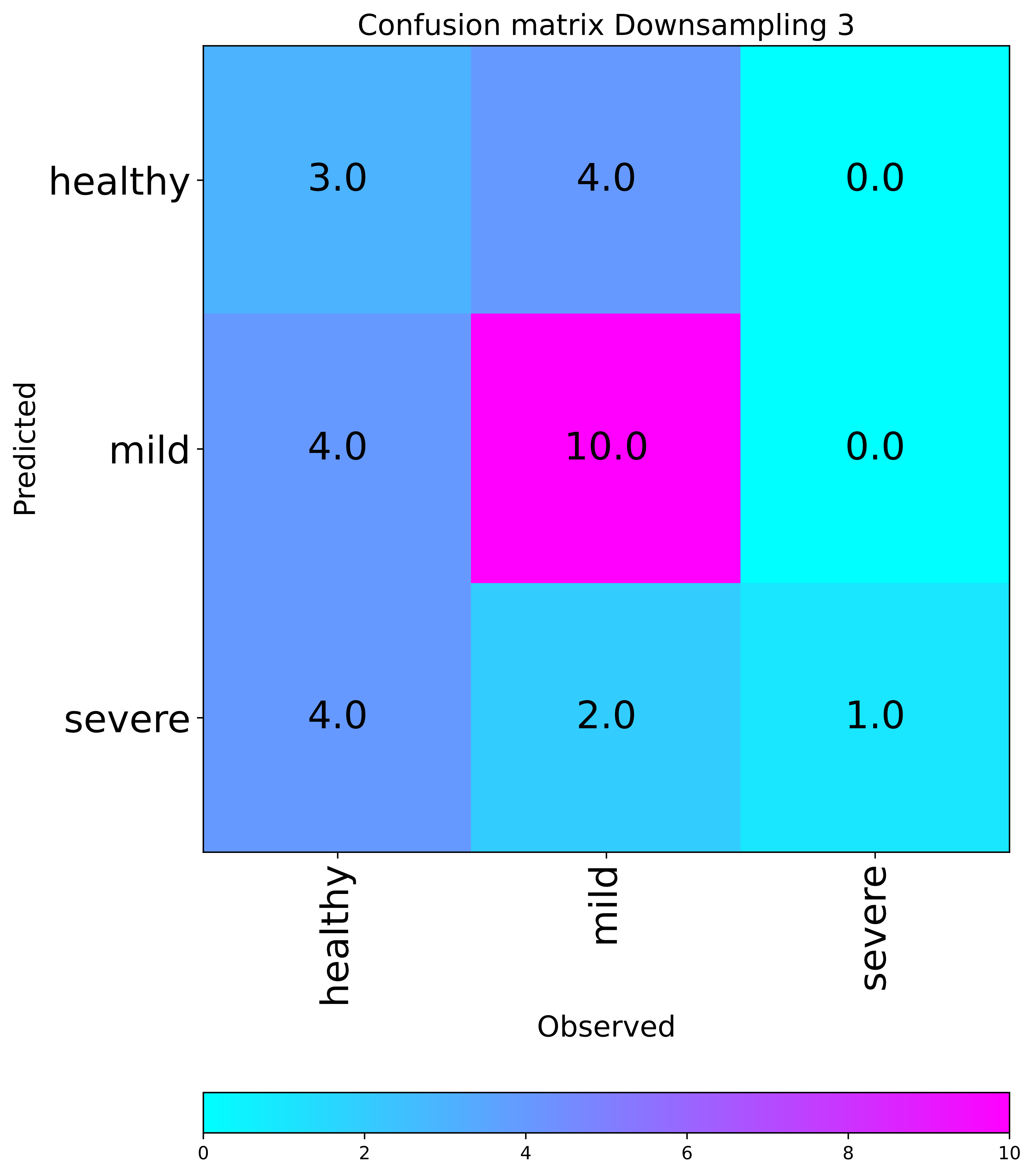

Confusion matrix Downsampling 3
Predicted
healthy
mild
severe
3.0
4.0
0.0
4.0
10.0
0.0
4.0
2.0
1.0
healthy
mild
severe
Observed
0
2
4
6
8
10
